# *Atg4b* Overexpression Extends Lifespan and Healthspan in *Drosophila melanogaster*

**DOI:** 10.3390/ijms24129893

**Published:** 2023-06-08

**Authors:** Yongxuan Li, Wei Zhang, Yunshuang Ye, Yinan Sun, Liping Yang, Guijun Chen, Kangning Chen, Sheryl Smith, Jumin Zhou

**Affiliations:** 1 Key Laboratory of Animal Models and Human Disease Mechanisms of Chinese Academy of Sciences/Key Laboratory of Healthy Aging Research of Yunnan Province, Kunming Key Laboratory of Healthy Aging Study, Kunming Institute of Zoology, Kunming 650223, China; liyongxuan20@mails.ucas.ac.cn (Y.L.); zhangwei21@mail.kiz.ac.cn (W.Z.); yeyunshuang@163.com (Y.Y.); jade_sun13@foxmail.com (Y.S.); yangliping@mail.kiz.ac.cn (L.Y.); chenguijun198692@163.com (G.C.); chenkangning@mail.kiz.ac.cn (K.C.); 2 Kunming College of Life Science, University of Chinese Academy of Sciences, Beijing 100049, China; 3 Biology Department, Arcadia University, Philadelphia, PA 19104, USA; smiths@arcadia.edu; 4 KIZ/CUHK Joint Laboratory of Bioresources and Molecular Research in Common Diseases, Kunming 650223, China

**Keywords:** *Atg4b*, longevity, healthy aging, *ATG4B*, cellular senescence

## Abstract

Autophagy plays important but complex roles in aging, affecting health and longevity. We found that, in the general population, the levels of *ATG4B* and *ATG4D* decreased during aging, yet they are upregulated in centenarians, suggesting that overexpression of ATG4 members could be positive for healthspan and lifespan. We therefore analyzed the effect of overexpressing *Atg4b* (a homolog of human *ATG4D*) in *Drosophila*, and found that, indeed, *Atg4b* overexpression increased resistance to oxidative stress, desiccation stress and fitness as measured by climbing ability. The overexpression induced since mid-life increased lifespan. Transcriptome analysis of *Drosophila* subjected to desiccation stress revealed that *Atg4b* overexpression increased stress response pathways. In addition, overexpression of *ATG4B* delayed cellular senescence, and improved cell proliferation. These results suggest that *ATG4B* have contributed to a slowdown in cellular senescence, and in *Drosophila*, *Atg4b* overexpression may have led to improved healthspan and lifespan by promoting a stronger stress response. Overall, our study suggests that *ATG4D* and *ATG4B* have the potential to become targets for health and lifespan interventions.

## 1. Introduction

Aging itself is the single most important risk factor for aging-related diseases, ranging from neurodegenerative diseases, cardiovascular diseases, to diabetes and cancer [1]. During the aging process, metabolism, immunity, fitness and many other bodily functions decline [2]. At cellular level, the accumulation of damaged macromolecules and organelles in cells that could not be removed in time due to declining autophagy function accelerate the aging process and increase disease risk [3]. Changes at the molecular level as a result of organismal aging include the upregulation of the mTOR pathway and immune pathway, and downregulation of MAPK, FOXO, lysosomal-autophagic signaling pathway and metabolic pathway [4,5,6]. These alterations in various types of cells in the organism lead to the aging of tissues and, ultimately the organism.

Autophagy, a highly conserved and essential cellular function, removes and degrades damaged organelles, macromolecules and intercellular debris. In a variety of organisms, autophagic activity decreases with age [7]. Lysosomal protein hydrolysis function also decreases with age, which impairs autophagic flux, thereby exacerbating cellular damage and promoting the development of aging-related diseases [8,9]. Autophagy-related genes *Atg2*, *Atg8a* and *bchs* are critical for both autophagy initiation and activity, and their expression decrease during the aging of *Drosophila* [10]. Likewise, the expression of autophagy-related protein beclin 1 (BECN1) decreases with age in rats [11]. The decrease in these proteins reduces cargo delivery to lysosomes, impairing autophagy, a major feature of organismal aging. Therefore, external interventions to improve autophagy to remove harmful macromolecules accumulated could be beneficial to improve individual health and prolong lifespan, and indeed, various anti-aging modalities seem to converge on autophagy [12]. Increased autophagic activity is also one of the necessary conditions for a variety of model animals to have a long-lived phenotype [13]. Overexpression of *HLH*-*30*, a master regulator of autophagy and lysosomal biogenesis, can extend worm lifespan [14], as well as the Golgi protein MON-2 can enhance autophagic activity by regulating Atg8/LGG-1, thereby allowing worm to live longer [15]. Overexpression of *ATG5* in mice also prolongs their lifespan [16]. In addition, specific overexpression of *Atg8* in the *Drosophila* nervous system also prolongs its lifespan [10].

However, excessive autophagy may lead to disturbances in cellular metabolism and accelerate aging [17]. Inhibiting autophagy levels in *Caenorhabditis elegans* (*C. elegans*) gut by high temperature prevents the emergence of aging-related phenotypes, [18] while decreased levels of the VPS-34–BEC-1–EPG-8 autophagy nucleation complex in *C. elegans* can prolong the lifespan of post-reproductive aged worms and improve its neuronal integrity [19]. The loss of SIRT1 activity is associated with multiple aspects of aging phenotype, yet SIRT1 is degraded by autophagy during aging [20]. The above information suggests that maintaining functional autophagy is essential for cellular and organismal health, and that autophagy, whether too low or too high, can lead to cellular defects and organismal functional decline.

The *ATG4* autophagic factors, including *ATG4A*, *4B*, *4C* and *4D* [21], are key cysteine protease enzymes processing *ATG8* during the formation of autophagesome, which couples *ATG8* to phosphatidylethanolamine on the autophagosome membrane [22]. Mutations in *ATG4D* led to *mAtg8* processing defects and degradation and transport in autophagesome [23]. Autophagy is induced by starvation stress, or the lack of growth factors to maintain the homeostasis of the intracellular environment during stress [7,24], which involves macroautophagy, a process regulated by the core autophagy machinery of key autophagy-related (ATG) proteins [25,26].

As *ATG4D* is one of the highly expressed autophagic factors in centenarians [27], we analyzed the effects of *ATG4D* on lifespan and healthspan via the overexpression of *ATG4D* in cells and *Atg4b* (a homolog of human *ATG4D*) in *Drosophila*. We found that the overexpression in flies improves resistance to oxidative stress, desiccation stress and climbing fitness; the overexpression also extends lifespan when *Atg4b* was induced since mid-life. Transcriptome data analysis at different time points of desiccation stress in *Drosophila* revealed that *Atg4b* overexpression accelerates cell renewal and increases the activation of stress response pathways. In cellular analyses, *ATG4B* overexpression slowed down cellular senescence. These findings suggest that ATG4 factors could improve health and lifespan.

## 2. Results

### 2.1. Transcript Levels of ATG4B and ATG4D Decreased during Aging

**Figure 1:**
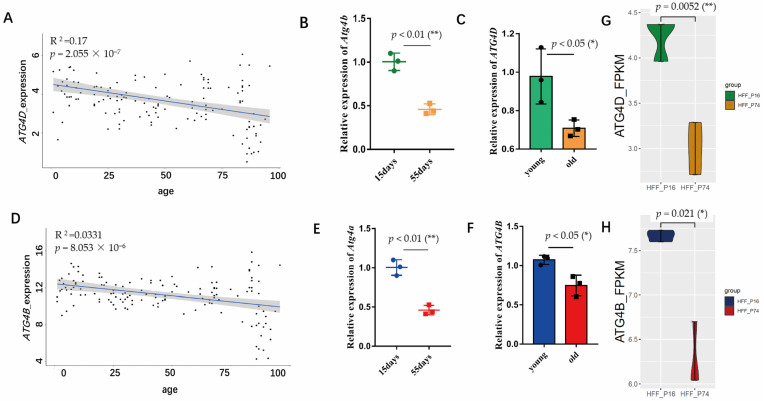
Changes in *ATG4D* and *ATG4B* expression with age. (**A**) Pearson correlation test between *ATG4D* expression and age (RNA-seq dataset of skin fibroblasts from 133 healthy subjects aged 1–94 years [28]), *p* = 2.055 × 10^−7^. (**B**) Reverse transcription (RT–qPCR) analyses of *Atg4b* in *Drosophila* at 15 days old and 55 days old (whole body for test), *n* = 3, two-tailed *t*-test, *p* < 0.01 (**). (**C**) Reverse transcription (RT–qPCR) analyses of *ATG4D* in IMR-90 cells of young and old, *n* = 3, two-tailed *t*-test, *p* < 0.05 (*). (**D**) Pearson correlation test between *ATG4B* expression and age (RNA-seq dataset of skin fibroblasts from 133 healthy subjects aged 1–94 years [28]), *p* = 8.053 × 10^−6^. (**E**) Reverse transcription (RT–qPCR) analyses of *Atg4a* in *Drosophila* at 15 days old and 55 days old (whole body for test), *n* = 3, two-tailed *t*-test, *p* < 0.01 (**). (**F**) Reverse transcription (RT–qPCR) analyses of *ATG4B* in IMR90 cells of young and old, *n* = 3, two-tailed *t*-test, *p* < 0.05 (*). (**G**) *ATG4D* expression based on FPKM from RNA-seq dataset of old human fibroblast cells (HFF_P74) and young cells (HFF_P16), *n* = 3, *t*-test, *p* = 0.0052 (**). (**H**) *ATG4B* expression based on FPKM from RNA-seq dataset of old human fibroblast cells (HFF_P74) and young cells (HFF_P16), *n* = 3, *t*-test, *p* = 0.021 (*).

Since we previously found upregulated levels of *ATG4* family transcripts in the peripheral blood transcriptome of centenarians [27], we subsequently examined the total RNA-seq dataset of skin fibroblasts from 133 healthy subjects aged 1–94 years [28] and found that *ATG4B* and *ATG4D* expression levels decreased with age via regression analysis of age and gene expression (Figure 1A,D), which suggested that the upregulated expression in centenarians was a specific trait for these long lifespan individuals. Next, we examined the *Drosophila* homologues of these genes, *Atg4b* (a homolog of human *ATG4D*) and *Atg4a* (a homolog of human *ATG4B*), respectively, and found that they also decreased from early life at day 15 to aged flies at day 55 (Figure 1B,E). Consistently, in human senescent embryonic lung fibroblast (IMR-90) cells, *ATG4B*, and *ATG4D* levels also lowered during cellular senescence (Figure 1C,F); similarly, it occurs in young and senescent human fibroblast (HFF) cells (Figure 1G,H) (online published data) [29,30]. These results suggest that *ATG4B* and *ATG4D* gene levels decline during normal organismal aging and cellular senescence.

### 2.2. Overexpression of ATG4B Delayed Cellular Senescence

**Figure 2:**
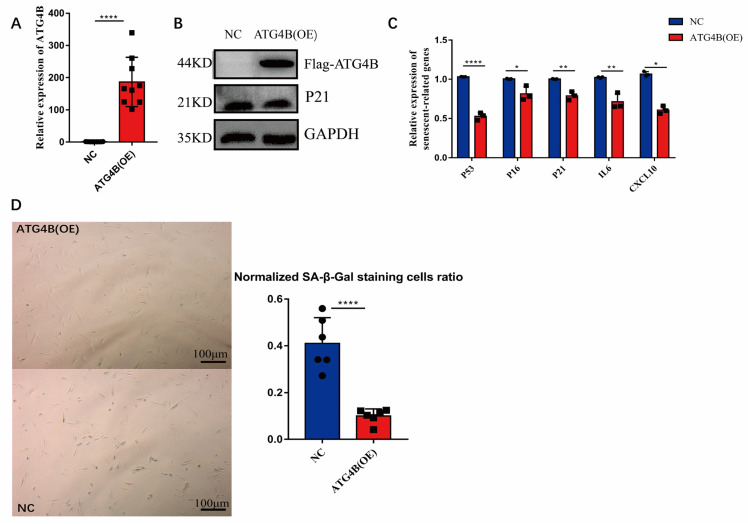
Overexpression of *ATG4B* in senescent cells and detection of cellular senescence phenotype. (**A**) Expression level of *ATG4B* in the overexpression group (ATG4B (OE)) and control group (NC) in IMR90 cells (P47) via qPCR, *n* = 3, two-tailed *t*-test, *p* < 0.0001 (****). (**B**) Flag-ATG4B, P21protein levels in IMR90 cells (P47) of *ATG4B* overexpression (ATG4B (OE)) and control group (NC) via Western-blot. (**C**) Transcript levels of aging-related marker genes *P53*, *P16*, *P21*, *IL6*, and *CXCL10* of overexpression *ATG4B* group (ATG4B (OE)) and control group (NC) in IMR90 cells (P47) via qPCR, *n* = 3, two-tailed *t*-test, *p* < 0.0001 (****), *p* < 0.05 (*), *p* < 0.01 (**), *p* < 0.01 (**), *p* < 0.05 (*). (**D**) Staining of β-galactosidase in IMR90 (P47) in the overexpression ATG4B (*ATG4B* (*OE*)) and control groups (NC), *n* = 6, two-tailed *t*-test, *p* < 0.0001 (****).

β-galactosidase is a widely used marker of cellular senescence [31] and high expression of *P53*, *P21*, *P16*, *IL6*, and *CXCL10* are molecular markers of cellular senescence [31,32,33,34]. To test whether ATG4 family factors prevent cellular senescence, we overexpressed *ATG4B* in IMR90 of passage 39 cells, and tested in passage 47 cells (Figure 2A,B), and found that the overexpression reduced β-galactosidase staining in the cells (Figure 2C). Similarly, reduced transcript levels of cellular senescence-related marker genes *P53*, *P21*, *P16, IL6,* and *CXCL10* (Figure 2D) and reduced P21 protein levels (Figure 2B) were also observed. This result is consistent with our previous analysis of *ATG4D* [27], and demonstrated that the overexpression of *ATG4B* also delay cellular senescence. 

### 2.3. Maintaining Early Atg4b Expression Levels during Late Life Extended Drosophila Lifespan

**Figure 3:**
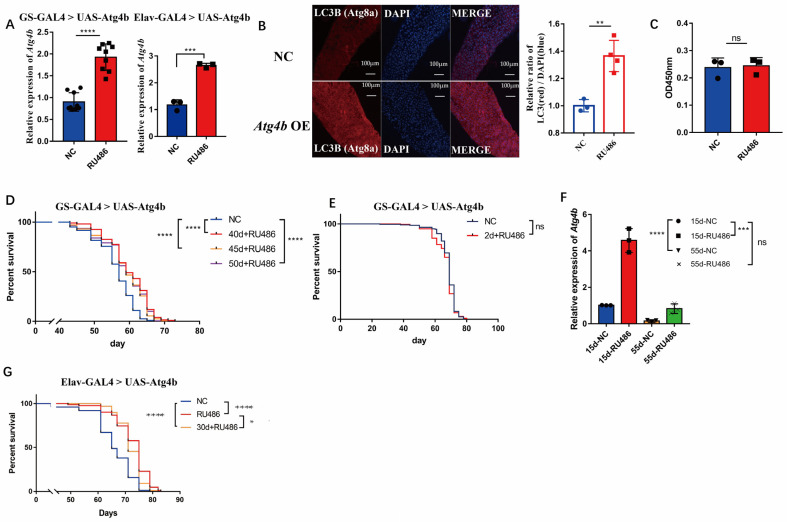
Survival of early and mid-late induced *Atg4b* overexpression in *Drosophila*. (**A**) Expression level of *Atg4b* in GS-GLA4 > UAS-Atg4b *Drosophila* group (RU486) and control (NC) group via qPCR (whole body for test), *n* = 9, two-tailed *t*-test, *p* < 0.0001 (****); Expression level of *Atg4b* in Elav-GLA4 > UAS-Atg4b *Drosophila* group (RU486) and control (NC) group via qPCR (only head for test), *n* = 3, two-tailed *t*-test, *p* < 0.001 (***). (**B**) Immunofluorescence detection of LC3 in the intestine of GS-GLA4 > UAS-Atg4b (*Atg4b* OE) and control group (NC), *n* = 3, two-tailed *t*-test, *p* < 0.01 (**). (**C**) *Drosophila* feeding assay in RU486 food and normal food, *n* = 3, two-tailed *t*-test, *p* > 0.05 (ns). (**D**) Survival of *Atg4b* overexpression induced by RU486 in *Drosophila* 40d (40d + RU486), 45d (45d + RU486), 50d (50d + RU486) and control group (NC), each group used flies *n* = 200, log-rank test, *p* < 0.0001 (****), *p* < 0.0001 (****), *p* < 0.0001 (****). (**E**) Survival of *Atg4b* overexpression induced by RU486 in *Drosophila* 2d (2d + RU486) and control group (NC), each group used flies *n* > 190, log-rank test, *p* > 0.05 (ns). (**F**) Expression levels of *Atg4b* in control *Drosophila* 15 days old (15d-NC), experimental group 15 days old (induced at 2 days old) (15d-RU486) and experimental group (induced at 40 days old) (55d-RU486) via qPCR (whole body for test), *n* = 3, two-tailed *t*-test, *p* < 0.0001 (****), *p* < 0.001 (***), *p* > 0.05 (ns). (**G**) Survival of *Atg4b* overexpression induced by RU486 in *Drosophila* nervous system 2d (RU486), 30d (30d + RU486) and control group (NC), each group used flies *n* > 75, log-rank test, *p* < 0.0001 (****), *p* < 0.0001 (****), *p* < 0.05 (*).

To test whether the overexpression of *Atg4b* (a homolog of human *ATG4D*) extends the lifespan in an organism, we analyzed *Drosophila Atg4b* overexpression in transgenic flies. The F1 generation from *da*-*GS*-*Gal4 Drosophila* crossed with *Atg4b* transgenic flies were induced by 100 μM RU486 to overexpress *Atg4b* in whole body, which was confirmed via R*T*-PCR (Figure 3A). The F1 generation from *Elav*-*Gal4 Drosophila* crossed with *Atg4b* transgenic flies were induced by 100 μM RU486 to overexpress *Atg4b* in the nervous system, which was confirmed via R*T*-PCR (Figure 3A). *Drosophila* with systemic overexpression of *Atg4b* detected elevated levels of Atg8a (a homologue of Human LC3B) protein downstream of Atg4 in the gut (Figure 3B), indicating that our system was working. Since food intake is a well-known factor affecting lifespan, and as an important control, we measured food intake in these flies, and found that there was no difference between the control and experimental groups (Figure 3C), therefore, the effects seen in the following experiments was solely due to *Atg4b* overexpression. 

The induction of *Atg4b* overexpression in whole body at 2 days of age had no effect on lifespan (Figure 3E). In contrast, the induction of *Atg4b* overexpression in whole body from mid-life, i.e., at 40, 45, or 50 days of age, prolonged lifespan in *Drosophila*. All groups of the median lifespan were extended from 57 to 59 days, which is an increase of 3.5% (Figure 3D). Given the different lifespan outcomes between early induction and mid-life inductions, we examined the expression levels of *Atg4b* in these groups, and found that the early induction group (induced at day 2, and measured at day 15) has as much as five times of *Atg4b* than the control group, while the mid-life induction group (induced at day 40, and measured at day 55) has a level similar to younger flies at day 15 (Figure 3F). This result suggests that excessive amount of *Atg4b* expression in *Drosophila* early in life was not beneficial for lifespan extension, while moderate induction since mid-life resulted in lifespan extension.

Since autophagy is also associated with neurodegenerative diseases [35], we tested whether *Atg4b* overexpression specifically in the *Drosophila* nervous system also improves lifespan. We performed specific induction of neurological overexpression of *Atg4b* in *Drosophila* at 2 days and 30 days of age and found that it prolonged *Drosophila* lifespan. The median lifespan was extended from 65 to 75 days, an increase of 15.38% (induced at day 2), and thereafter the median lifespan was extended from 65 to 71 days, an increase of 9.23% (induced at day 30) (Figure 3G). This suggests that CNS is more sensitive to low levels of *ATG4* during normal aging and induced *Atg4b* in the nervous system may enhance autophagy in the brain and thus prolong *Drosophila* lifespan.

### 2.4. Atg4b Overexpression Improved Drosophila Healthspan

Healthspan measures how long an individual or an organism stays healthy. Factors that increase fitness and stress resistance are important for healthspan and lifespan extension. Important measurement of healthspan in *Drosophila* is a number of fitness and stress resistance tests include resistance to oxidative stress, desiccation stress and fitness to assess healthspan. Resistance to oxidative stress mainly reflects the DNA repair capacity and ROS metabolism, while the resistance to starvation and desiccation depends on autophagy and regulation of metabolism [36,37,38,39,40,41]. Here, flies with *Atg4b* overexpression in whole body induced at 2 days old were assayed at 20 days of age, while the flies induced at 40 days of age were assayed at day 55.

**Figure 4:**
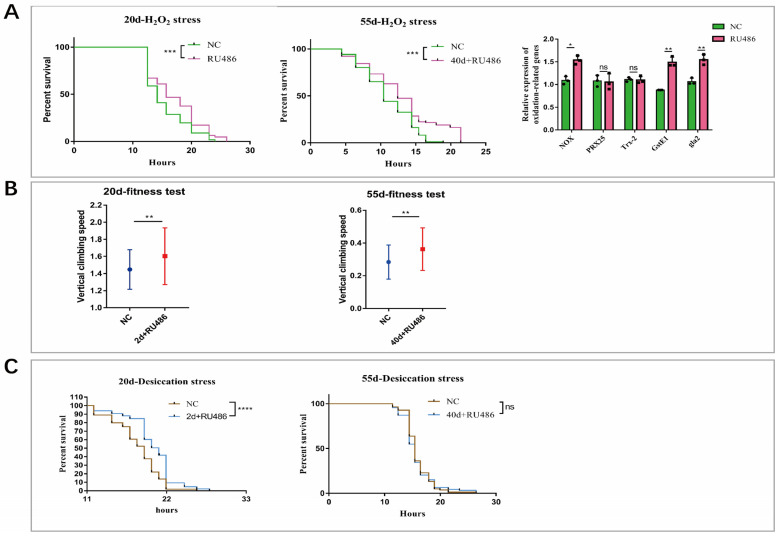
Stress survival of *Drosophila* in *Atg4b* overexpression group and control group. (**A**) Survival of 30% hydrogen peroxide treated 20-day-old *Drosophila* (induced at 2 days of age) (RU486), 55-day-old *Drosophila* (induced at 40 days of age) (40d + RU486) and control (NC), each group used flies *n* > 60, log-rank test, *p* < 0.001 (***), *p* < 0.001 (***); transcript levels of oxidative stress-related genes *NOX, PRX25*, *Trx*-*2*, *GstE1*, and *gla2* via qPCR in 30% hydrogen peroxide treated induced and control groups after 10 h, *n* = 3, two-tailed *t*-test, *p* < 0.05 (*), *p* > 0.05 (ns), *p* > 0.05 (ns), *p* < 0.01 (**), *p* < 0.01 (**). (**B**) Vertical climbing speed of 20-day-old *Drosophila* (induced at 2 days of age) (2d + RU486), 55-day-old *Drosophila* (induced at 40 days of age) (40d + RU486) and control (NC), each group used flies *n* > 80, two-tailed *t*-test, *p* < 0.01 (**), *p* < 0.01 (**). (**C**) Survival of 20-day-old *Drosophila* (induced at 2 days of age) (2d + RU486), 55-day-old *Drosophila* (induced at 40 days of age) (40d + RU486) and control (NC) at desiccation stress, each group used flies *n* > 80, log-rank test, *p* < 0.0001 (****), *p* > 0.05 (ns).

Oxidative stress level increases with age and affects the normal function of various tissues, leading to many chronic diseases associated with aging, such as diabetes, cardiovascular and cerebrovascular and skeletal muscle diseases [42,43]. Hydroxyl radicals (OH) are highly reactive towards lipids, proteins and DNA and, at high concentrations, are severely detrimental to cell survival [44]. The resulting OS is an imbalance between the formation of reactive oxygen species (ROS) and antioxidant defense mechanisms. To test whether *Atg4b* protects flies against oxidative stress, we added 30% hydrogen peroxide (H_2_O_2_) to the medium, and found that the induction of *Atg4b* overexpression in *Drosophila* at both 2 and 40 days of age improved the resistance to oxidative stress. The median survival time increased from 19.42 h to 23.42 h, an increase of 20.6% (induced at day 2, and tested at day 20), and later it increased by 19.16% (induced at day 40, and tested at day 55) (Figure 4A). RT–PCR detection after oxidative stress treatment was applied for 10 h showed that the expression of anti-oxidative stress genes increased in the *Atg4b* overexpression group. Specifically, *Atg4b* enhanced the activation of several genes in the oxygen free radical scavenging pathways, including *NOX*, *GstE1* and *gla2*, resulting in enhanced antioxidant capacity in *Drosophila*. (Figure 4A).

Desiccation stresses the organism with cell dehydration which could damage the phospholipid bilayer of cell membranes [45], concentrate electrolytes and damage the structure of DNA and multiprotein complexes [46,47]. Desiccation survival relays on the proper metabolic and homeostasis regulation. During this test, flies were subjected to desiccation stress at 25 °C by incubating in vials without water and food. *Drosophila* with induced *Atg4b* overexpression at 2 days of age had increased desiccation resistance, with the median survival extended from 25.9 h to 32.3 h, an increase of 24.79%, however, unlike the oxidative stress test, *Drosophila* with *Atg4b* overexpression at 40 days of age showed no difference in desiccation resistance from the controls (Figure 4C). Thus, increased expression of *Atg4b* promoted resistance to desiccation stress during early, but not late lifespan.

With the accumulate dysfunctional mitochondria and protein aggregates resulting from aging, muscle function declines, which is manifested in decreased muscle strength and total muscle mass, and this could be measured via fitness tests [12,48]. To test whether *Atg4b* also improve fitness in flies, we placed the flies in empty culture tubes, tied to a horizontal board together with a ruler, and filmed to record the climbing process to the top of the tube after they were shaken down to the bottom of the tube. Climbing ability was measured by climbing speed. We found that *Atg4b* overexpression in *Drosophila* at both early life of 2 days and mid-life from 40 days of age improved the climbing ability of *Drosophila.* The vertical climbing speed is increased from 1.447 to 1.602 mm/s, an increase of 10.7% (induced at day 2, and tested at day 20), and then it is increased from 0.2837 to 0.3623 mm/s, an increase of about 21.7% (induced at day 40, and tested at day 55) (Figure 4B).

From the results of oxidative stress, fitness and desiccation stress experiments, we conclude that overexpressing *Atg4b* in *Drosophila* could improve the antioxidant capacity and enhance muscle motility, with the exception that *Atg4b* overexpression enhances *Drosophila* desiccation resistance only when induced at an early stage but not later in life.

### 2.5. Atg4b Regulated Metabolism and Cell Cycle, and Facilitated Switch to Stress Response Pathways

**Figure 5:**
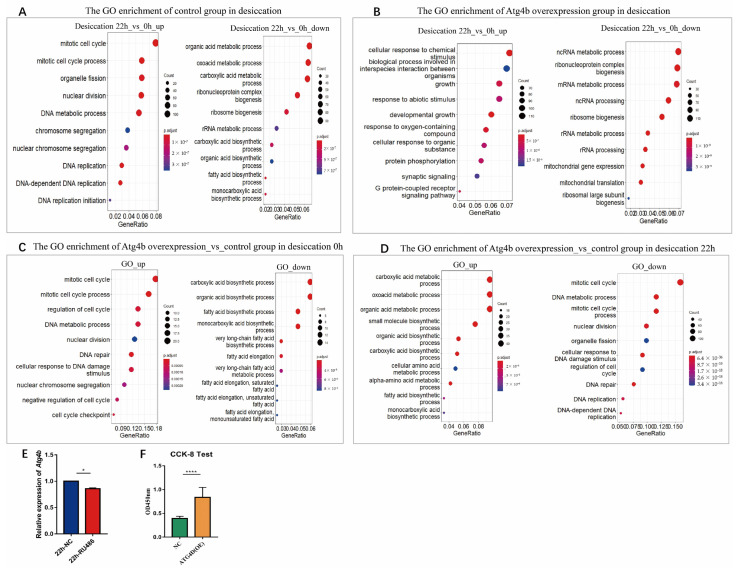
Transcriptome analysis of desiccation stressed 20-day-old *Drosophila*. Flies were induced *Atg4b* overexpression at day 2, and desiccation applied at 20 days of age for 22 h. Control (untreated, or 0 h were included). Top 10 biological processes are shown. (**A**) The GO enrichment of control group in desiccation. (**B**) The GO enrichment of *Atg4b* overexpression group in desiccation, top 10 biological processes are shown. (**C**) The GO enrichment of *Atg4b* overexpression group compared with control group in desiccation 0 h; 10 biological processes are shown. (**D**) The GO enrichment of *Atg4b* overexpression group compared with control group in desiccation 22 h; 10 biological processes are shown. (**E**) Expression levels of *Atg4b* in the experimental group (22h-RU486) and control groups (22h-NC) after 22 h of desiccation treatment via qPCR, *n* = 3, two-tailed *t*-test, *p* < 0.05 (*). (**F**) Cell proliferation for *ATG4D* overexpression (*ATG4D* (OE)) cells (IMR90 P47) detected via cell counting assay, *n* = 8, two-tailed *t*-test, *p* < 0.0001 (****).

In the above experiments, we observed apparently contrasting results between lifespan and desiccation tests when *Atg4b* was induced in whole body early in life versus mid-life, i.e., early induction promoted desiccation resistance, yet had no beneficial effects on lifespan, whereas mid-life induction extended lifespan but had no effects on desiccation resistance. In an attempt to explain the apparent contradictory results, we checked the expression of *Atg4b* at 22 h after desiccation stress was applied, and found that the level of *Atg4b* in overexpression group (induced at day 2 and tested at day 20) quickly dropped to a lower level than control flies (Figure 5E), suggesting that the level of ATG4 is subject to rapid down regulation in desiccation stress, and the additional high level of *Atg4b*, which only exist when induced early, provided this protection from stress. The day 40 induction, in contrast, could not induce as high a level as day 2 induction, and thus did not offer protection in desiccation stress.

Next, we conducted a transcriptome analysis on these flies. We found that in the control group, the 22 h of desiccation treatment increased cell cycle, endosomal transport and protein phosphorylation related genes, while it downregulated organic acid metabolic process, ribosome biogenesis and mitochondrial translation related genes (Figure 5A). In comparison, in *Atg4b* overexpression flies, the treatment upregulated cellular response to chemical stimulus, metamorphosis and regulation of intracellular signal transduction, while downregulating mRNA/ncRNA metabolic process, ribosome biogenesis and mitochondrial gene expression (Figure 5B). These results suggest that *Atg4b* overexpression increased the activation of stress response pathways, and as a result, may have improved their resistance.

We next compared the overexpression group with controls under these treatment conditions. In 0 h (untreated), overexpressing *Atg4b* reduced metabolism but increased cell cycle and related transcription pathways (Figure 5C); when desiccation stress was applied for 22 h, stress response pathways, cell communication pathways increased, while cell cycle and related transcriptional pathways become downregulated (Figure 5D). As the overexpressed *Atg4b* level dropped rapidly following the desiccation test, which coincided with decrease in cell cycle related genes, we speculate that the overexpressed *Atg4b* may have increased cell cycle activity. Consistent with this notion, in IMR90 of passage 47 cells, overexpressing *ATG4D* indeed enhanced cell activity and accelerated cell proliferation (Figure 5F). 

## 3. Discussion

We studied the ATG4 factors, as *ATG4B*, and *ATG4D* were highly expressed in centenarians [27], yet their levels are progressively lower in the general population [28] (Figure 1A,D). First, we found that overexpression of *ATG4D* and *ATG4B* in IMR90 of passage 47 cells can delay cellular senescence (Figure 2, [27]), suggesting that the overexpression of ATG4B andATG4D in centenarians could be a positive factor for longevity, rather than a result of extreme aging. 

The *p*-values in the regression analysis of ATG4B/4D with age were very small despite the low R^2^ (Figure 1A,D), indicating a trend toward significance despite the high variability of the sample data between individuals. In particular, there were very significant differences in the distribution at later stages. Since this dataset was not sampled continuously, we hypothesize that the strong variation in expression at the high end of the horizontal axis (around 80–90 years) may divide the population into two categories: those who continue to live long lives (e.g., centenarians) and those who have reached a late stage of life where high expression of ATG4B and ATG4D may help achieve longevity. In *Drosophila*, we added 100 μM of RU486 to the control flies and found that it did not affect lifespan (Supplementary Figure S3). The overexpression of *Atg4b* increased resistance to oxidative stress, desiccation stress and climbing ability (Figure 4), and the overexpression induced since mid-life increased lifespan (Figure 3D). These suggest that maintaining *Atg4b* expression levels at mid-late stages of *Drosophila* life is beneficial for extending lifespan and improving health. It suggests that the reduction in *Atg4b* due to aging is counterbalanced to achieve a delay in the aging process. 

Transcriptome analysis during desiccation stress verified that *Atg4b* confers resistance to desiccation stress likely by quickly switching from promoting cell cycle to activating stress resistance pathways (Figure 5). This study suggests that the overexpression of *ATG4D* in centenarians could be a positive factor for human health and lifespan rather than a consequence of extreme aging.

Activation of the autophagic pathway is beneficial to extend lifespan, but excessive activation may also lead to accelerated aging [17,27], thus, all overexpression autophagy factors are not positive for health and lifespan. Our results show that the timing of overexpression is also essential, as the induction of *Atg4b* overexpression at 2 days of age has no effect on *Drosophila* lifespan, but there is a transient phase of substantial increase in mortality throughout mid-life (Figure 3E). However, the induction of *Atg4b* overexpression at 40 days of age was able to prolong *Drosophila* lifespan (Figure 3D). The higher expression level of *Atg4b* in the day 2 induction group than in the day 40 group (Figure 3F), suggests that too high a level of *Atg4b* was not conducive to the extension of lifespan. Additionally, since the expression of *Atg4b* decreased with age (Figure 1B), maintaining its level later in life is more important than excessive early expression for lifespan extension.

The lysosomal-autophagic pathway also plays an important role in neurodegenerative diseases [49,50,51,52], as we previously showed that *Drosophila dNAGLU* (a homolog of human *NAGLU*, a lysosomal enzyme) reduced Aβ42 deposition in AD *Drosophila* and human U251-APP cells by enhancing lysosomal function [53]. At the same time, in a recently published paper, it was found that bi-allelic loss-of-function variants in *ATG4D* is the cause of syndromic neurodevelopmental disorders [54]. Here, we found that specific overexpression of *Atg4b* in the *Drosophila* nervous system at both 2 and 30 days of age prolonged lifespan, with the 2-day-old induction group living longer than the 30-day-old induction group (Figure 3G), suggesting that the CNS could be the main tissue where ATG4D exert their functions, and maintaining higher levels of *Atg4b* expression in the *Drosophila* nervous system throughout the life cycle is more conducive to lifespan extension than the induced overexpression in the entire body. 

Not all members of ATG4 family act the same way during aging. The expression of *ATG4B* and *ATG4D* decreased with aging (Figure 1A,D), while the expression of *ATG4A* did not significantly correlate with age and *ATG4C* increased with aging (Supplementary Figure S1A,B). In senescent cells, both *ATG4B* and *ATG4D* overexpression delayed cellular senescence (Figure 2) [27], which may be due to the inconsistent splicing activity among various ATG4 proteins [55].

Transcriptome data offered potential mechanism on how Atg4b may promote health and longevity. During desiccation stress, metabolic pathways were attenuated, while stress pathway genes were upregulated in the *Atg4b*-overexpressing group, suggesting that these flies can better devote resources to combating stress. The autophagy pathway could be activated by the MAPK pathway cascade to regulate cell proliferation, differentiation, and stress adaptation [56]. Damaged stress-activated autophagy pathway can regulate the cell cycle by downregulating P53 [57,58]. It is possible that enhanced cell proliferation by overexpressed *ATG4D* in senescent cells (Figure 5F) and the improved desiccation resistance in *Drosophila* due to overexpressed *Atg4b* may upregulate cell cycle-related pathways through the activation of autophagy (Figure 4C and Figure 5), which in turn enhances the ability of cellular health, cell renewal and improves desiccation resistance. 

When desiccation was applied, *Atg4b*-overexpressing *Drosophila Atg4b* transcript levels dropped to lower levels than the control (Figure 5E), and a dramatic increase in the death of *Drosophila* (survival percentage) was observed after *Atg4b* levels dropped, from more than 40% to less than 10% (Figure 4C). We speculate that *Atg4b* plays an important role in regulating the on- or off-state transition of life activity processes. Similarly, in oxidative stress test, where the overexpression of *ATG4B* and *ATG4D* enhanced cellular resistance to H_2_O_2_ in our in vivo experiments (Supplementary Figure S2), the additional *Atg4b* may also have increased upregulation of stress response pathways, resulting in stronger oxidative stress resistance. This mechanism linking *Atg4b* and a faster response to stress may be due to an overall improvement in cellular function, as we have shown that *ATG4D* promotes the cell cycle in senescent cells. However, it could also be due to an immediate, unknown mechanism. 

Taken together, our results revealed an important link between ATG4 factors and healthspan and lifespan, and offered ATG4, in particular *ATG4D* and *ATG4B*, as potential target for intervention of neurodegenerative diseases. Further study on ATG4 agonist and *Atg4b*-specific expression in the *Drosophila* nervous system (Figure 3G) could allow us to understand how *Atg4b* agonists could potentially elevate endogenous *Atg4b* expression to prolong healthspan and lifespan.

## 4. Materials and Methods

### 4.1. Drosophila Stocks and Construction of the Transgenic Flies

For the construction of transgenic flies, we used molecular cloning technology to insert *Atg4b* into the UAS promoter-driven vector *pUAST*-*attB* to generate *pUAST*-*attB*-*Atg4b* plasmid, which was then microinjected into *86F (III) 6110* transgenic strain (gift of Sun Yat-Sen University) with the attP site inserted near 86F. *Atg4b* transgenic flies were obtained via standard transgenic. The *Atg4b* transgenic flies were then backcrossed with *w^1118^* flies for 5 generations to remove the background. The da-*GS*-*Gal4* flies (gift from Sichuan Agricultural University) expressed steroid-activated GAL4 in the whole body. ELAV (gift from Sichuan Agricultural University) flies expressed steroid-activated GAL4 in the nervous system. The *w^1118^* flies were wild-type flies. 

### 4.2. Husbandry and Lifespan Analysis

The larval stage of all the strains of *Drosophila* were fed standard cornmeal-yeast medium (agar 7.6 g/L H_2_O, soy flour 13.5 g/L H_2_O, corn flour 92.7 g/L H_2_O, malt flour 61.8 g/L H_2_O, yeast 22.9 g/L H_2_O, syrup 206.1g/L H_2_O, and propionic acid 6.4mL/L H_2_O). During the adult experimental stage, flies were fed sucrose-yeast (SY) food (yeast 100 g/L H_2_O, agar 15 g/L H_2_O, sugar 50 g/L H_2_O, propionic acid 3 mL/L H_2_O, and 10% methyl nuns 30 mL/L H_2_O).

The *Atg4b* transgenic flies were crossed with driver lines *GS*-*Gal4* and *ELAV*-*Gal4*. In the experimental group, the F1 generation flies were fed SY food supplemented with RU486 (*w*/*v*, 100 µM; Sigma-Aldrich, St. Louis, MO, USA, CAS 84371-65-3) to induce *Atg4b* overexpression in the whole body and nervous system. Additionally, in the control group, F1 generation flies were fed with SY food without RU486 but supplemented with the same dose of ethanol.

After hatching, the larvae were mated in standard cornmeal-yeast medium for at least 48 h, and female flies were selected for experimental research. We used *Drosophila* for lifespan statistics with 10 flies/tube, and each tube contained an appropriate amount of SY food, at least 20 tubes per experimental condition. All stocks were maintained at 25 °C and 60% humidity, 12 h light, and 12 h dark cycle. The flies were transferred to fresh food every 2 days, during which the number of dead and escaped flies was recorded. All lifespan data were analyzed using the log-rank test.

### 4.3. Health Test Assays

A total of 10 flies/tube were used for the test, with at least 20 tubes per experimental condition. For the desiccation test, the flies were placed in an empty tube without medium, and the number of dead flies was counted every 2 h. For the H_2_O_2_ oxidative stress test, circular filter paper was placed at the bottom of the culture tube, 50 μL of 6% glucose solution containing 30% H_2_O_2_ was added, then the flies were put into the tube, and the number of dead flies was counted every 2 h [59]. All survival data were analyzed using the log-rank test. For the climbing test, 10 flies/tube were used for the test, with at least 10 tubes per experimental condition. We calculated the average speed of flies climbing from the bottom of the tube to the top to evaluate the climbing ability of flies. The climbing data were analyzed using the *t*-test.

### 4.4. Immunofluorescence

*Drosophila* intact intestine was dissected and directly fixed by compression, permeabilized for 15 min, closed for 30 min, incubated overnight at 4 degrees with primary antibody (anti-LC3I, Abcam, Cambridge, UK, ab192890), and subsequently incubated with Alexa Fluor 594-conjugated secondary antibody (ThermoFisher, Waltham, MA, USA, A-11012) for 1 h at room temperature. The slides were mounted with mounting medium with DAPI (Abcam, Cambridge, UK, ab104139). Imaging was then performed using an ultra-high-resolution laser confocal microscope (Zeiss, Oberkochen, Germany, LSM880), and mean fluorescence intensity analysis was performed via Image J 1.8.0_172 (64-bit).

### 4.5. qPCR

RNA was extracted with RNAiso Plus (TaKaRa, Kusatsu, Japan, RNAA00250). Then, RNA was reverse transcribed into cDNA using Prime Script TMRT reagent kit with gDNA Eraser (TaKaRa, Kusatsu, Japan, RR047Q), and qR*T*-PCR was performed using the Fast Start Universal SYBR Green Master (ROX) (Roche, Basel, Switzerland, 04913850001) and gene-specific primers. All the above experimental operations were performed according to the manufacturer’s procedures. Gene expression levels were calculated using the comparative C_T_ method. All primers used are listed in (Supplementary Table S1).

### 4.6. Western Blot

The total protein was extracted with the RIPA lysis buffer (Beyotime, Haimen, China, P0013B), and the protein extracts were added to the lanes of SDS-PAGE gels and transferred to PVDF membranes, and the membranes were incubated with antibodies. The primary antibodies were anti-P21 (CST, Boston, MA, USA, 2947S), anti-FLAG (Abcam, Cambridge, UK, ab1162) and anti-LC3I (Abcam, Cambridge, UK, ab192890). The secondary antibodies were goat anti-mouse IgG (Invitrogen, Waltham, MA, USA, 31430) and goat anti-rabbit IgG (Invitrogen, Waltham, MA, USA, 31460). The internal reference antibody was anti-GAPDH (Abcam, Cambridge, UK, ab8245). 

### 4.7. ATG4 Expression Analysis

Changes in *ATG4* expression with increasing age in the population [28] were simulated using a simple linear regression model. Pearson’s correlation test was used to evaluate the association between gene expression and age through the correlation test function in the R platform. The data that support the findings of this study (RNA-seq datasets) are available in the Gene Expression Omnibus (GEO), accession numbers GSE113957 (human fibroblasts), and data on the expression of ATG4B/4D in HFF cells are available in GSE63577. 

### 4.8. Cells Culture

IMR90 were cultured in Minimum Essential Medium (MEM basic) (C11095500BT, Gibco, Billings, MT, USA) containing 10% fetal bovine serum (35-076-CV, Gibco) and 1% penicillin/streptomycin (15140-122, Gibco) in a 37 °C/5% CO_2_ incubator.

Cell passage was performed using a Count Star instrument (MS7-H550-Pro, SCILOGEX) for live cell counting, followed by equal and uniform plate spreading, and all cell experiments were performed after cell passage to the wall.

### 4.9. Cells and Transfection

IMR90 cells was a gift from Xudong Zhao. *ATG4B* overexpression plasmid was constructed using the pTomo-CMV-loxP-mRFP-loxP-EGFP lenti-viral vector and was then transduced into IMR90 cells. Stably transfected cells were selected via fluorescence screening.

### 4.10. Cell Proliferation Analysis

For CCK-8 assays, after 48 h of treatment, 10 μL CCK-8 solution (Yeasen, Shanghai, China, #40203ES60) was added to each well. The plates were incubated in an incubator for 3 h, and then absorbance at 450 nm was determined.

### 4.11. SA-β-Galactosidase Staining

NC and ATG4B (OE) groups were stained with β-galactosidase after equal amounts of cells were evenly applied to the wall (each group had six replicate experiments). β-Galactosidase staining was performed using a senescence-associated β-galactosidase staining kit (Beyotime, Shanghai, China). Cells were washed three times with PBS and fixed with 4% paraformaldehyde for 15 min at room temperature. The cells were then incubated overnight at 37 °C in the dark with the working solution containing 0.05 mg/mL X-gal.

We then divided each group of cell plate into four areas, and randomly selected five visual fields for shooting in each area. Next, the proportion of stained cells in the total number of cells were calculated, and then the average value was taken as the final percentage of each experimental stained cell. Approximately 300 cells were photographed per disc, and data from six repeated experiments from two groups were tested using *t*-tests.

### 4.12. Bioinformatics Analysis

The polyA-enriched RNA-sequencing (RNA-seq) libraries were prepared for sequencing using the Illumina HiSeq 6000 platform. FastQC v0.11.9 (https://github.com/s-andrews/FastQC/releases/tag/v0.11.9 accessed on 26 April 2023) was used for quality control of all raw data. The clean data were then aligned to the *D. melanogaster* genome in UCSC (dm6) using Hisat2 v2.2.1 [60]. The FeatureCounts [61] was used to count reads of genes. DESeq2 [62] for experimental and control groups for differential gene were screened in R. Differentially expressed genes were subjected to Gene Ontology (GO) enrichment analysis using the ClusterProfiler v4.2.1 package [63] and visualized using the “ggplot2” package in R. Pearson’s correlation test was used to evaluate the association between gene expression and age samples through the cor.test function in the R platform. Significance was determined a priori at a Benjamini–Hochberg (BH)-corrected *p*-value of <0.05.

### 4.13. Statistical Analysis

All survival data were analyzed using the log-rank test (Includes: Natural Life Expectancy Statistics, Stress Survival Statistics). The climbing data, QPCR data and β-galactosidase staining data were analyzed using the *t*-test. DESeq2 for differential gene screening, Significance was determined a priori at a Benjamini–Hochberg (BH)-corrected *p*-value of <0.05.

## Data Availability

The source data underlying Figure 5A–D were deposited in BIG Sub (accession numbers: PRJCA014675). All other data are available from the corresponding author.

## Supplementary Materials

The supporting information can be downloaded at: https://www.mdpi.com/article/10.3390/ijms24129893/s1.
