# Supplementary material for: Atg4b Overexpression Extends Lifespan and Healthspan in Drosophila melanogaster
Source: Int J Mol Sci. 2023 Jun 8;24(12):9893. doi: 10.3390/ijms24129893 (PMC10298381; doi:10.3390/ijms24129893)
Supplement: Supplementary file 1 [file ijms-24-09893-s001.zip › ijms-2396517-supplementary.pdf]

Supplementary Figures

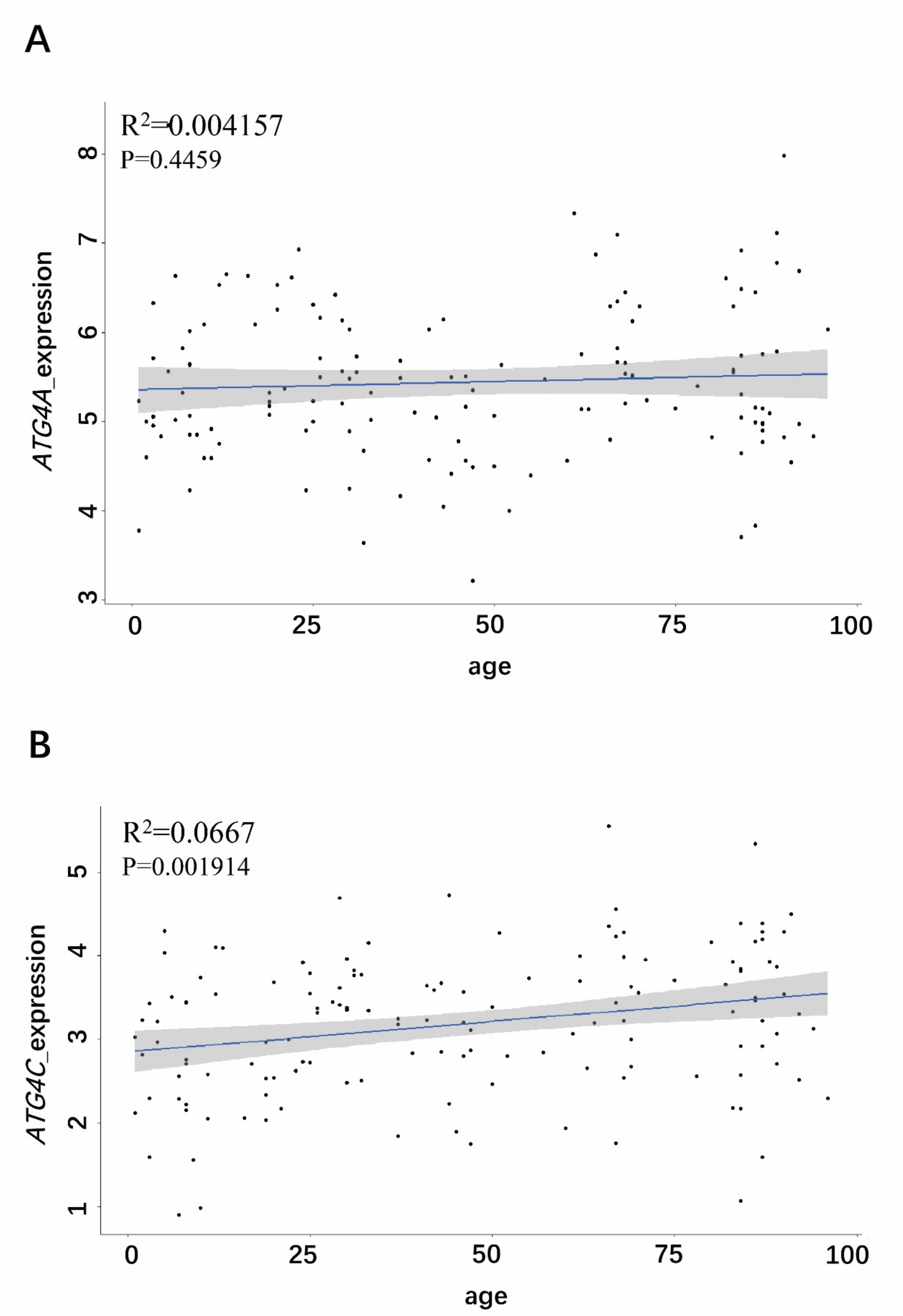

Supplementary Figure S1. Changes of *ATG4A* and *ATG4C* expression with age.

(A) Pearson correlation test between *ATG4A* expression and age (RNA-seq dataset of skin fibroblasts from 133 healthy subjects aged 1-94 years [28]), P=0.4459.

(B) Pearson correlation test between *ATG4C* expression and age (RNA-seq dataset of skin fibroblasts from 133 healthy subjects aged 1-94 years [28]), P=0.001914.

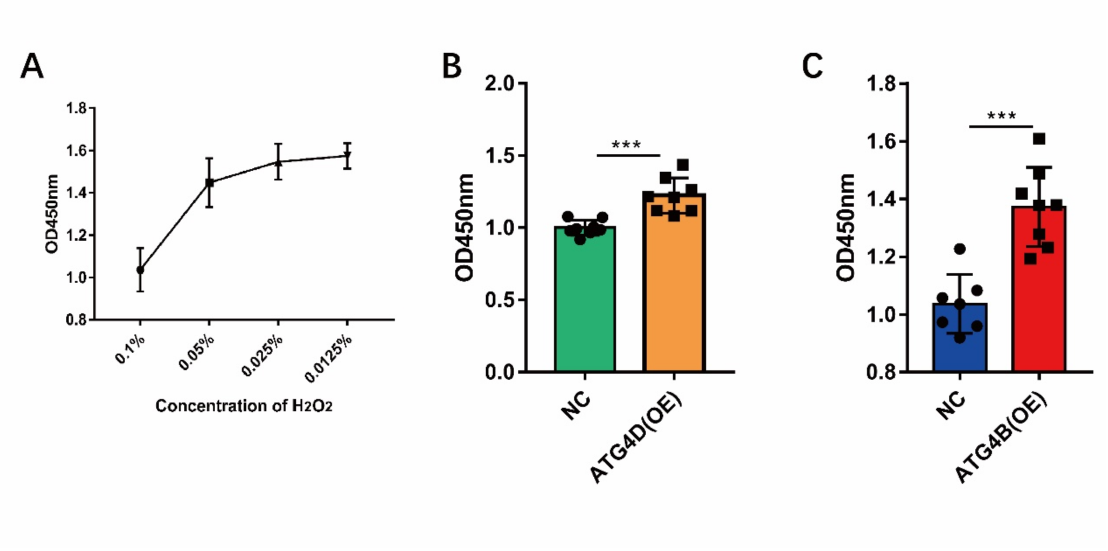

Supplementary Figure S2. Cell proliferation curve for *ATG4B* and *ATG4D* overexpression after 0.05% hydrogen peroxide treatment compared to the control group.

(A) Cell proliferation for control cells (IMR90 P47) treated with different concentrations of hydrogen peroxide by cell counting assay, n=8.

(B) Cell proliferation for *ATG4D* overexpression (*ATG4D*(OE)) cells (IMR90 P47) after 0.05% hydrogen peroxide treatment by cell counting assay, n=8, two-tailed t-test, $p<0.001$(***).

(C) Cell proliferation for *ATG4B* overexpression (*ATG4B*(OE)) cells (IMR90 P47) after 0.05% hydrogen peroxide treatment by cell counting assay, n=8, two-tailed t-test, $p<0.001$(***).

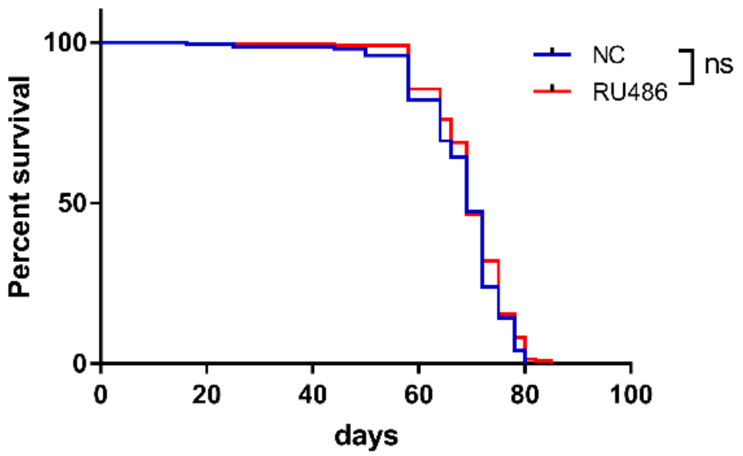

Supplementary Figure S3. Survival of Drosophila in RU486 induction and non-induction.

RU486-induced UAS-GAL4 flies (RU486) and non-RU486-added controls (NC), flies used per group n=200, log-rank test, p>0.05 (ns).

Supplementary Table S1 QPCR primer information

Species	Primer name	Primer sequences
Drosophila melanogaster	Atg4a-F	TCCTCGCTAGTGCAGTTGG
Drosophila melanogaster	Atg4a-R	CTTCTTTCCCAGGACCCAGAC
Drosophila melanogaster	Atg4b-F	GACCATTGTAGAGGGTAGCCG
Drosophila melanogaster	Atg4b-R	CGATGAATCGTCTGTATGGGG
Drosophila melanogaster	Actin5c-F	AAGCTGTGCTATGTTGCCCT
Drosophila melanogaster	Actin5c-R	ATTCCCAAGAACGAGGGCTG
Drosophila melanogaster	Trx-2-F	ACAACGTCGTCGTCCTGAAG
Drosophila melanogaster	Trx-2-R	AACTCTTCGACCTTGACGCC
Drosophila melanogaster	gla2-F	CGATTTCCGGACGTGATTGC
Drosophila melanogaster	gla2-R	TCTGATCGGAGTGACCCAGT
Drosophila melanogaster	GstE1-F	GCCAAGTCGGATGAGCTGTA
Drosophila melanogaster	GstE1-R	CGTTTATCCAAAACGGGCGG
Drosophila melanogaster	PRX25-F	ACTGTCACCGATGAAGAGGC
Drosophila melanogaster	PRX25-F	AGTAGTTGTCAGTGGTGCGG
Drosophila melanogaster	NOX-F	GTGGGGGATCAACTGGTAGC
Drosophila melanogaster	NOX-R	TGAGCGCCAAAGATGTGACT
Homo sapiens	ATG4B-F	GGTGTGGACAGATGATCTTTGC
Homo sapiens	ATG4B-R	CCAACTCCCATTTGCGCTATC
Homo sapiens	ATG4D-F	GGAACAACGTCAAGTACGGTT
Homo sapiens	ATG4D-R	CTCGCCCTCGAAACGGTAG
Homo sapiens	P53-F	CAGCACATGACGGAGGTTGT
Homo sapiens	P53-R	TCATCCAAATACTCCACACGC
Homo sapiens	P16-F	GATCCAGGTGGGTAGAAGGTC

Homo sapiens	P16-R	CCCCTGCAAACTTCGTCCT
Homo sapiens	P21-F	TGTCCGTCAGAACCCATGC
Homo sapiens	P21-R	AAAGTCGAAGTTCCATCGCTC
Homo sapiens	IL6-F	AGACAGCCACTCACCTCTTCAG
Homo sapiens	IL6-R	TTCTGCCAGTGCCTCTTTGCTG
Homo sapiens	CXCL10-F	GCTCTACTGAGGTGCTATGTTC
Homo sapiens	CXCL10-R	GGAGGATGGCAGTGGAAGTC
Homo sapiens	ATG5-F	AGAAGCTGTTTCGTCCTGTGG
Homo sapiens	ATG5-R	AGGTGTTTCCAACATTGGCTC
Homo sapiens	LAMP-F	TCTCAGTGAACTACGACACCA
Homo sapiens	LAMP-R	AGTGTATGTCCTCTTCCAAAAGC
Homo sapiens	P62-F	GGAGAAGAGCAGCTCACAGCCA
Homo sapiens	P62-R	CCTTCAGCCCTGTGGGTCCCT
Homo sapiens	BECN-F	GGTGTCTCTCGCAGATTCATC
Homo sapiens	BECN-R	TCAGTCTTCGGCTGAGGTTCT
Homo sapiens	18S-F	GTAACCCGTTGAACCCCATT
Homo sapiens	18S-R	CCATCCAATCGGTAGTAGCG
